# Age Group-Specific Improvement of Vertebral Scoliosis after the Surgical Release of Congenital Muscular Torticollis

**DOI:** 10.1055/a-2168-4606

**Published:** 2024-02-07

**Authors:** Jong Min Choi, Seong Hoon Seol, Jae Hyun Kim, Chan Min Chung, Myong Chul Park

**Affiliations:** 1 Department of Plastic and Reconstructive Surgery, Hallym Sacred Heart Hospital, College of Medicine, Hallym University, Seoul, Korea

**Keywords:** torticollis, neck muscles, surgical procedures, operative, scoliosis

## Abstract

**Background** Congenital muscular torticollis (CMT) is a common musculoskeletal disorder in children. Secondary scoliosis can occur in patients with CMT; however, the extent of inclination and improvement of scoliosis after surgical correction of CMT have not been adequately studied. In this study, we aimed to evaluate and measure the improvement in vertebral tilting after surgical correction according to age at the time of surgery.

**Methods** Between June 2007 and January 2020, 831 patients with CMT underwent sternocleidomastoid release. Among them, 426 patients were enrolled, and their medical records were retrospectively reviewed. Ultimately, 210 patients available for radiological evaluation and analysis were enrolled in this study. The patients were divided into four groups according to age at the time of surgery to determine the relationship between age and changes in scoliosis.

**Results** Our findings showed an improvement in scoliosis in all age groups after surgery. The results for follow-up after 1 year confirmed long-term improvement in vertebral tilting. The degree of improvement in scoliosis was significantly higher in the younger age group than in patients aged 18 years or older.

**Conclusion** The effect of surgical release on scoliosis was significant in all age groups. The findings of this study suggest that CMT should be corrected before the age of 3 years to ensure an optimal surgical mitigation of scoliosis. Furthermore, in cases of neglected CMT, surgical release should be actively attempted because there is significant improvement.

## Introduction

Congenital muscular torticollis (CMT) is a rare form of musculoskeletal disorders that manifests during early infancy. It limits neck movement due to sternocleidomastoid (SCM) muscle contracture. Its prevalence is reported to be 0.3 to 2.0%, it is usually congenital in origin, and, rarely develops from an acquired disease. 1 2 Although the etiology of CMT remains unclear, intrauterine crowding, malposition, and birth trauma have been identified as contributing factors. CMT occurs due to SCM fibrosis, and SCM contracture limits neck movement in such cases. 1 3

Approximately 90% of neck movement limitation can be improved with stretching exercises; however, symptoms that persist even after continuous physical therapy can cause changes in the shape of the head, asymmetry of the face, and secondary scoliosis. Earlier surgical correction for disease intractable to physical therapy is generally recommended; however, controversy regarding the appropriate time for surgical intervention persists. 4 Few studies have shown excellent clinical results regarding neck movement and cosmetic correction after surgical treatment of neglected CMT in adults and children over 5 years of age. 5

Secondary scoliosis can occur in patients with neglected CMT; however, there are no adequate studies on this issue. 4 5 6 Furthermore, reports on the developmental outcomes of surgical release are limited. Regular measurement and evaluation of vertebral development after surgical correction are essential. In this study, we aimed to evaluate and measure the improvement in vertebral scoliosis after surgical correction of CMT according to the age of patient at the time of surgery.

## Methods

### Patients

We retrospectively reviewed medical records of patients who underwent surgical treatment of the SCM muscle for CMT between June 2007 and January 2020. Eight hundred and thirty-one patients with CMT underwent SCM release. The exclusion criteria were (1) torticollis of unknown etiology or neurologic causes, (2) osseous torticollis, (3) history of surgical release on the same side, (4) history of injection of botulinum toxin into the affected SCM, (5) individuals who did not undergo plain radiography of the whole spine, in preoperative and postoperative periods, and (6) less than 6 months of follow-up. Of the 426 patients who satisfied the abovementioned criteria, 210 were available for radiological evaluation and analysis and were enrolled as participants in this study.

The patients were divided into four groups according to age at the time of surgery to determine the relationship between age and changes in scoliosis. We divided the patients into age groups of 0–1, 1–3, 3–18, and >18 years. In general, 1–3 years of age is well-known indication for surgery; however, in severe CMT, physical therapy is not expected to help at all, and surgery is sometimes performed before 1 year of age. Therefore, patients younger than 3 years were divided into age groups of 0–1 and 1–3 years. Among the neglected cases over 3 years of age, patients were divided into age groups of 3–18 years with ongoing bone growth, and >18 years with complete bone growth.

### Surgical Technique and Postoperative Care

A senior author (M.C.P.) performed the surgical procedure, mostly involving unipolar release. This surgical technique combines complete tight band release and segmental resection of a fibrous mass. Under general anesthesia, the patient was placed in the supine position, with the head turned away from the affected side. An incision was made transversely 1.5–3.0 cm above the clavicle on the affected side. After protecting the external jugular vein and dividing the platysma in-line with the incision, the deep cervical fascia was penetrated, and the two heads of the SCM muscle were identified. The tight bands and muscles were divided, and abnormal fibrous bands were excised. After the surgical procedure, a soft neck collar was immediately placed in the neutral neck position and worn for 3 weeks. Physiotherapy was started 2 weeks after surgery and continued for 6 months to regain the neck's full passive range of motion (PROM). Muscle strengthening exercises are strongly recommended for children older than 4 years.

### Evaluation

**Fig. 1:**
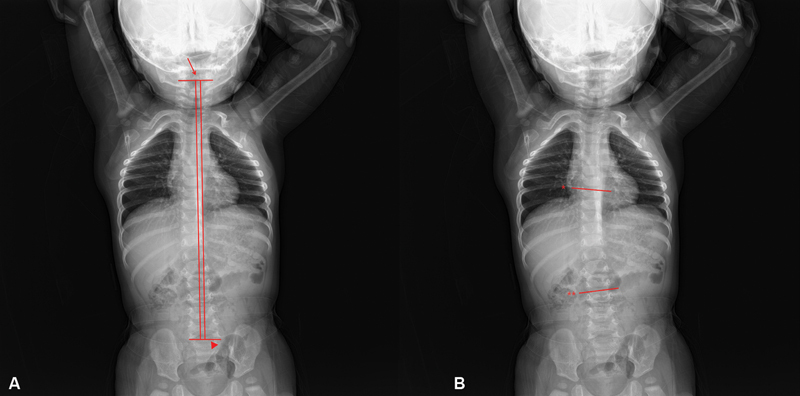
Spinal deformity measurement in terms of CA and LS values. ( **A** ) LS is defined as the distance between the central sacral vertical line and the central vertical line of the C2 vertebral body (arrow: C2 vertebral body, arrowhead: sacral upper margin). ( **B** ) Cobb angle is defined as the angle formed by two perpendicular lines between the superior endplate of the proximal end vertebra and the inferior endplate of the distal end vertebra (asterisk: superior endplate of the proximal end vertebra, two asterisks: inferior endplate of the distal end vertebra). CA, Cobb angle; LS, lateral shift.

Informed consent was taken from patients for the evaluation of imaging studies. All postoperative time referred to in this article means the latest follow-up period. Anteroposterior (AP) plain radiographs were taken of the whole spine to measure the lateral shift (LS) and the Cobb angle (CA). To evaluate cervical scoliosis, LS was measured from AP radiographs of the entire spine. LS was defined as the distance between the central sacral vertical line and the central vertical line of the C2 vertebral body 4 ( Fig. 1A ). The CA was measured from an AP plain radiograph of the entire spine to evaluate the severity of secondary vertebral scoliosis. The CA was defined as the angle formed by two perpendicular lines between the superior endplate of the proximal end vertebra and the inferior endplate of the distal end vertebra 7 ( Fig. 1B ). The end vertebra in scoliosis is defined as the most tilted vertebra. So, proximal end vertebra is the most tilted vertebra above the apex, and distal end vertebra is the most tilted vertebra below the apex.

The preoperative and postoperative LS and CA values were compared. The percentage changes between preoperative and postoperative periods were compared by age groups. The percentage change was defined as ([Post − Pre]/Pre] × 100[%]). The significance of age and time, and the interaction effect of age and time were also statistically measured. All analyses were conducted for the total group and the group with 1-year follow-up.

### Statistical Analysis

R language version 3.3.3 (R Foundation for Statistical Computing, Vienna, Austria) and the T&F program ver. 3.0 (YooJin BioSoft, Korea) were used for all the statistical analyses. *p* -values less than 0.05 were considered statistically significant. Data were presented as medians (interquartile range [IQR]), and the Wilcoxon signed-rank test was used to compare the LS (mm) and CA measurements before and after operation. The percentage changes between preoperative and postoperative periods were compared using the Kruskal–Wallis H test. Bonferroni correction was used for post hoc analysis. Because the data did not follow a normal distribution, a statistical test was performed using a nonparametric method such as the Wilcoxon signed-rank test and Kruskal–Wallis H test, and the statistics are expressed as median (IQR).

Repeated-measures analysis of variance (ANOVA) was performed to test the respective effects of age and time variables when the other variable was controlled. The age variable was defined as the age group (0–1, 1–3, 3–18, >18 years) and the time variable was defined as the pre- and postoperative time of CMT. The interaction effect between age and time was also confirmed using ANOVA.

## Results

### Comparison of Lateral Shift and Cobb Angle Values Before and After Operation

**Fig. 2:**
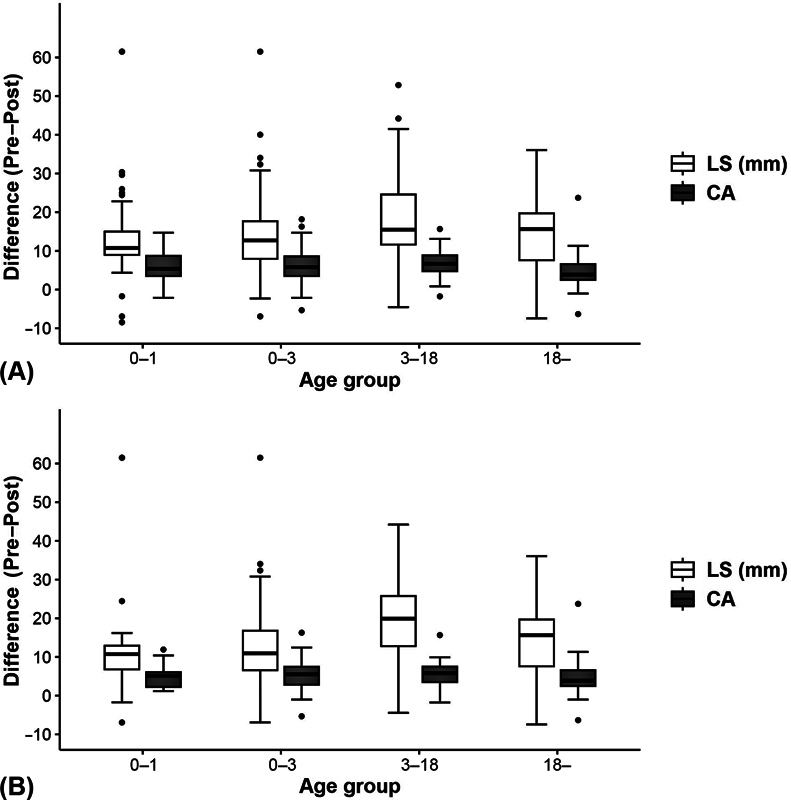
Comparison of LS and CA values before and after operation. The distribution of (Pre − Post) values for each variable is displayed as a box plot. The straight line inside the box represents the median, and the bottom/top of the box represents the interquartile range (values corresponding to 25–75% of the sample). ( **A** ) Comparison between pre- and postoperative values for all patients. ( **B** ) Comparison between pre- and postoperative values for patients at 1-year follow-up. CA, Cobb angle; LS, lateral shift.

There was a statistically significant difference in LS and CA between pre- and postoperative time points in all groups (total and each age group; *p* < 0.001). In the total group, the postoperative LS was significantly lower than the preoperative LS—median 8.4 mm (IQR, 5.43–12.15 mm) versus median 22.2 mm (IQR, 16.32–32.13 mm). The postoperative CA was lower than the preoperative CA—median of 3.96 (IQR, 2.68–6.28) versus median of 9.97 (IQR, 7.15–14.16). Both LS and CA variables significantly decreased after surgery compared to those before surgery in all age groups ( Table 1 ; Fig. 2A ).

**Table 1:** Comparison of lateral shift and Cobb angle before and after operation for all patients

Variable	Subgroup	*N* (%)	Pre	Post	Pre–Post	*p* -Value
LS (mm)	Total	210 (100.0)	22.20 (16.32–32.13)	8.40 (5.43–12.15)	12.91 (8.59–19.76)	<0.001
	0–1	57 (27.1)	18.62 (16.04–28.59)	8.40 (4.82–12.51)	10.77 (8.71–15.09)	<0.001
	1–3	73 (34.8)	21.45 (15.29–28.51)	8.45 (4.82–11.34)	12.50 (7.75–17.82)	<0.001
	3–18	52 (24.8)	26.88 (17.69–39.34)	7.42 (6.35–11.04)	15.50 (11.48–24.61)	<0.001
	18–	28 (13.3)	23.25 (19.15–38.93)	10.97 (5.10–19.80)	15.63 (7.52–19.92)	<0.001
CA	Total	210 (100.0)	9.97 (7.15–14.16)	3.96 (2.68–6.28)	5.77 (3.31–8.76)	<0.001
	0–1	57 (27.1)	9.65 (6.95–13.93)	3.87 (2.37–5.96)	5.36 (3.41–8.79)	<0.001
	1–3	73 (34.8)	9.85 (7.12–13.89)	3.78 (2.53–5.66)	5.77 (3.45–8.71)	<0.001
	3–18	52 (24.8)	11.51 (8.97–15.46)	4.17 (2.88–7.13)	6.66 (4.67–8.84)	<0.001
	18–	28 (13.3)	8.78 (5.93–15.30)	5.40 (3.31–7.83)	3.85 (2.31–7.52)	<0.001

Abbreviations: CA, Cobb angle; LS, lateral shift.

Both LS and CA values significantly decreased after operation ( *p* < 0.001).

There were statistically significant differences in LS and CA between pre- and postoperative periods in all groups that were followed up 1 year after surgery ( *p* < 0.001; Table 2 ; Fig. 2B ).

**Table 2:** Comparison of lateral shift and Cobb angle before and after operation for 1-year follow-up patients

Variable	Subgroup	*N* (%)	Pre	Post	Pre–Post	*p* -Value
LS (mm)	Total	113 (100.0)	22.26 (16.05–30.80)	8.77 (6.27–12.20)	12.91 (7.18–19.80)	<0.001
	0–1	20 (17.7)	17.49 (16.03–25.32)	8.93 (7.06–12.42)	10.75 (6.61–12.92)	<0.001
	1–3	45 (39.8)	19.61 (12.66–26.45)	8.62 (4.79–11.73)	10.59 (5.36–16.80)	<0.001
	3–18	20 (17.7)	29.31 (18.08–42.04)	7.78 (6.89–10.27)	19.89 (12.78–29.01)	<0.001
	18–	28 (24.8)	23.25 (19.15–38.93)	10.97 (5.10–19.80)	15.63 (7.52–19.92)	<0.001
CA	Total	113 (100.0)	9.21 (6.15–12.43)	3.63 (2.42–6.16)	4.86 (2.55–7.31)	<0.001
	0–1	20 (17.7)	9.21 (5.63–10.36)	3.50 (1.92–6.20)	5.11 (2.23–6.23)	<0.001
	1–3	45 (39.8)	8.75 (6.15–10.87)	3.34 (1.85–4.27)	5.53 (2.81–7.51)	<0.001
	3–18	20 (17.7)	10.02 (7.34–13.30)	3.43 (2.73–5.68)	5.79 (3.39–8.24)	<0.001
	18–	28 (24.8)	8.78 (5.93–15.30)	5.40 (3.31–7.83)	3.85 (2.31–7.52)	<0.001

Abbreviations: CA, Cobb angle; LS, lateral shift.

Both LS and CA values significantly decreased after operation ( *p* < 0.001).

### Comparison of Change of Lateral Shift and Cobb Angle among Age Ranges

**Fig. 3:**
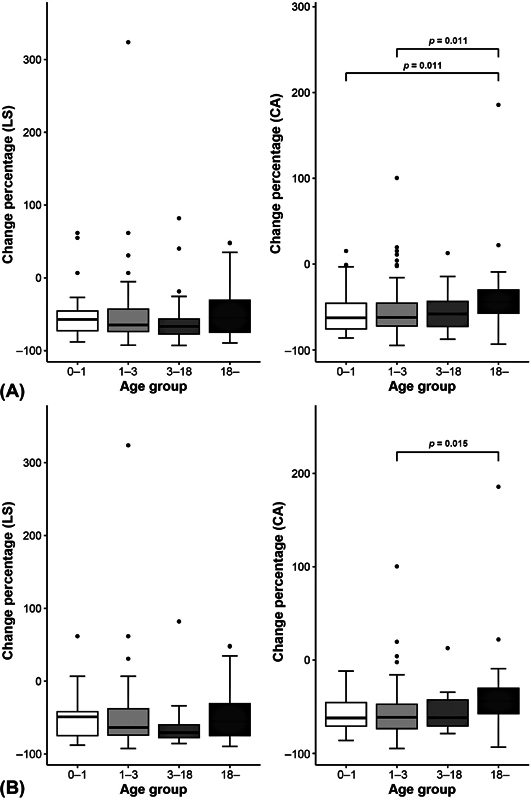
Comparison of changes of LS and CA values among patients in different age ranges. The distribution of change (%) values for each variable is displayed as a box plot. The straight line inside the box represents the median, and the bottom/top of the box represents the interquartile range (values corresponding to 25–75% of the sample). ( **A** ) Comparison of changes of LS and CA values among age ranges for all patients. CA change rate was significantly different between the 0-to-1-year-old group and 18-years-and-older group and between 1-to-3-year-old group and 18-years-and-older group ( *p* < 0.05). ( **B** ) Comparison of changes of LS and CA values among age ranges for patients at 1-year follow-up. CA change rate was significantly different between the 1-to-3-year-old group and the 18-year-old group ( *p* < 0.05). CA, Cobb angle; LS, lateral shift.

The results were analyzed to compare the percentages of LS and CA changes before and after surgery among the four age groups. LS showed no significant difference between the age groups ( *p* = 0.217) whereas CA showed significant differences between the age groups ( *p* = 0.009) in the total group. The changes in CA (%) in the 0-to-1-year-old group and 18-years-and-older group were −62.65 (IQR, −75.49 to −45.66) and −44.11 (IQR, −59.01 to −28.97), respectively, the rate of change was significantly greater in the 0-to-1-year-old group ( *p* = 0.011). Additionally, between the 1-to-3-year-old group and the 18 years-and-older group, with values of −62.12 (IQR, −72.84 to −45.25) and −44.11 (IQR, −59.01 to −28.97), respectively, the rate of change was significantly greater in the 1-to-3-year-old group ( *p* = 0.011). As a result of the post hoc test, the LS change rate did not show a significant difference between age groups, but the CA change rates were significantly different between the 0-to-1-year-old group and 18-years-and-older group and between 1-to-3-year-old group and 18-years-and-older group ( Table 3 ; Fig. 3A ).

**Table 3:** Comparison of changes of lateral shift and Cobb angle values according to age ranges for all patients

Age range	*N* (%)	Change percentage (LS)	Change percentage (CA)
0–1	57 (27.1)	−57.19 (−72.67 to −44.68)	−62.65 (−75.49 to −45.66)
1–3	73 (34.8)	−64.72 (−73.97 to −42.80)	−62.12 (−72.84 to −45.25)
3–18	52 (24.8)	−66.69 (−77.69 to −55.93)	−58.09 (−72.83 to −43.41)
18–	28 (13.3)	−55.14 (−75.13 to −30.25)	−44.11 (−59.01 to −28.97)
*p* -Value	–	0.217	0.009
0–1 vs. 1–3	–	1.000	1.000
0–1 vs.3–18	–	0.383	1.000
0–1 vs.18–	–	1.000	0.011
1–3 vs.3–18	–	1.000	1.000
1–3 vs.18–	–	1.000	0.011
3–18 vs.18–	–	0.533	0.089

Abbreviations: CA, Cobb angle; LS, lateral shift.

CA change rates were significantly different between the 0-to-1-year-old group and 18-years-and-older group and between 1-to-3-year-old group and 18-years-and-older group ( *p* < 0.05).

The results were analyzed to determine whether there was a difference in the rate of change among the four age groups before and after surgery in the 1-year follow-up group. As a result of analyzing the change in the CA (%) in the 1-to-3-year-old group and 18 years-and-older group, with −61.29 (IQR, −73.62 to −46.82) and −44.11 (IQR, −59.01 to −28.97) values respectively, the rate of change was significantly greater in the 1-to-3-year-old group. The rate of change in LS did not show a significant difference between the age groups. However, the CA change rate was significantly different between 1-to-3-year-old group and 18 years-and-older group ( Table 4 ; Fig. 3B ).

**Table 4:** Comparison of changes of lateral shift and Cobb angle values according to the age of patients for 1-year follow-up patients

Age range (years)	*N* (%)	Change percentage (LS)	Change percentage (CA)
0–1	20 (17.7)	−49.00 (−76.26 to −41.05)	−62.03 (−71.23 to −45.14)
1–3	45 (39.8)	−63.59 (−74.41 to −32.29)	−61.29 (−73.62 to −46.82)
3–18	20 (17.7)	−70.60 (−78.59 to −57.82)	−61.73 (−71.17 to −41.13)
18–	28 (24.8)	−55.14 (−75.13 to −30.25)	−44.11 (−59.01 to −28.97)
*p* -Value	–	0.400	0.018
0–1 vs.1–3	–	1.000	1.000
0–1 vs.3–18	–	1.000	1.000
0–1 vs.18–	–	1.000	0.132
1–3 vs.3–18	–	0.686	1.000
1–3 vs.18–	–	1.000	0.015
3–18 vs.18–	–	0.948	0.223

Abbreviations: CA, Cobb angle; LS, lateral shift.

CA change rate was significantly different between the 1-to-3-years-old group and the 18-years-and-older group ( *p* < 0.05).

### Analysis of Effect of Group and Time Variable and Interaction between Group and Time

**Fig. 4:**
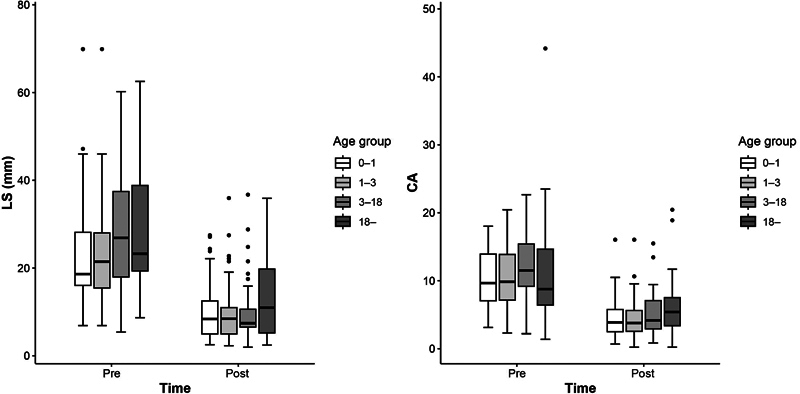
Time trend of LS and CA values according to age ranges. The distribution of LS and CA values for each variable is displayed as a box plot. The straight line inside the box represents the median, and the bottom/top of the box represents the interquartile range (values corresponding to 25–75% of the sample). CA, Cobb angle; LS, lateral shift.

The effect of age variable was not significant for both LS ( *p* = 0.055) and CA ( *p* = 0.098) when the time variable was controlled. However, the effect of time was significant of both LS and CA ( *p* < 0.001) when the age variable was controlled. The interaction between age and time was not statistically significant for either the LS or CA ( Table 5 ). The time trends of LS and CA according to age group are shown in a box plot ( Fig. 4 ).

**Table 5:** *p* -Values for the effect of age and time in repeated-measures analysis of variance

	LS	CA
Factor	*p* -Value	*p* -Value
Age	0.055	0.098
Time	<0.001	<0.001
Age × time (interaction)	0.062	0.350

Abbreviations: CA, Cobb angle; LS, lateral shift.

The effect of time was significant for both LS and CA values ( *p* < 0.001) when the age variable was under control. The interaction between age and time was not statistically meaningful ( *p* > 0.05).

## Discussion

CMT is the congenital musculoskeletal abnormality which may be accompanied by other congenital diseases such as mental retardation, second and third cervical spine fusion, and spina bifida. 8 9 As the number of CMT is small, there has been insufficient discussion and consensus regarding the timing of surgical correction. Decisions to perform surgical correction of CMT at an early age should be made with caution. This is because such early surgical intervention involves the risk of general anesthesia in newborns, as well as the challenging environment of the surgical field. 10 11 The surgical environment of the infant head and neck is very narrow and small. The underlying structures such as internal jugular vein, spinal accessory nerve, or thoracic duct are highly susceptible to injury, especially in early and very fibro-contracted SCM. 12 13 Though, it is also difficult to wait until patients are older because secondary disability due to CMT can be exacerbated. Facial asymmetry, one of the most well-known secondary disorders of CMT, occurs due to prolonged unilateral contracture of the SCM muscle and several reports have revealed that early release of SCM (before the age of 5 years) can facilitate better correction of craniofacial asymmetry. 14 15 16 17

There are insufficient reports about timing of surgical correction of CMT in terms of CMT-related scoliosis. Scoliosis can occur as the result of a neglected CMT. The release of the contracted SCM muscle can be beneficial not only for deviated posture correction of the neck, but also for bony scoliosis. Improvement in scoliosis is greater when surgery is performed before the age of 15 years. 18 Another study reported significant effectiveness of surgical release for spinal deformities. The effectiveness of surgical release for spinal deformities has been already proven. 19

Our findings showed a significant improvement in scoliosis in all age groups after surgery. The follow-up results for 1 year confirmed long-term improvement of scoliosis. To establish the optimal timing of surgery for CMT considering the improvement of scoliosis, we compared the rate of improvement of scoliosis among the age ranges. It was confirmed that the severity of secondary vertebral scoliosis, which the CA value can identify, improved more in patients in 0–3 years of age group than in those older than 18 years. In addition, even in the case of a 1-year follow-up period, the degree of improvement in scoliosis was higher in the group aged 1–3 years compared to that in the group aged 18 years or older. Considering the improvement in scoliosis, patients with CMT who do not respond to physical treatments are advised to undergo correction before the age of 3 years if surgery is possible. The rate of change in CA was−62.65 and −62.12%, in the results of the groups of 0–1 and 1–3 years, showing better improvement.

Of course, the improvement in scoliosis was less than that of patients in the early stages, but neglected patients also showed remarkable improvement. As mentioned earlier, surgery at the age of 3 years or younger showed the best results; however, CMT at other ages should be corrected, regardless of age. Repeated-measures ANOVA confirmed that the time variable (pre- and postoperative time) had a meaningful effect on LS and CA values when the age variable was controlled, and the interaction between time and age was not significant. This indicates that LS and CA values improved after surgery, regardless of age. Hence, our results show that, in patients with early-stage CMT, as well as in patients with neglected CMT, surgical release has a beneficial effect on scoliosis. Our results can establish another basis for the timing of surgery, even in patients with neglected CMT, given that invasive surgical releases can reduce spinal deformities in patients aged >18 years. Improvement in scoliosis measured using CA increased significantly from the age of 3 years at the time of surgery. This might be due to the greater potential for scoliosis remodeling after surgery in young patients.

Physiotherapy, including muscle strengthening exercises, plays a significant role in the treatment of CMT and can also impact the treatment of scoliosis. 3 Physiotherapy and warm pack application are recommended for achieving neck's full PROM for 6 months after surgery. Additionally, muscle strengthening exercises are strongly recommended from the age of 4 to 5 years. The loss of SCM muscle due to surgical release does not result in significant weakness of the neck. However, exercises may help provide support for the cervical spine by strengthening other muscles. Muscle strengthening exercises are expected to improve CMT-related scoliosis by enhancing the stability of the cervical spine and aiding in posture correction. Nevertheless, since sufficient analysis and discussion of the effects have not been made yet, additional studies are required.

This study has some limitations. The LS and CA values were measured using AP radiograph of the entire spine. The quality of AP radiography is lower in children with poor coordination than in adults. Moreover, depending on the radiology technician who obtained the AP radiograph, there may have been gaps in the scanning range or posture. Patients with poor quality or significant differences were excluded from the data collection stage; however, it was difficult to completely eliminate variables that occurred during the imaging process.

In conclusion, quantitative evaluation of AP plane radiographs showed that the improvement in scoliosis—evaluated using LS and CA—was the most significant in 1–3 years of age. The degree of improvement decreased with the age at the time of surgery; however, the improvement was still significant. The improvement in scoliosis due to CMT surgery was significantly beneficial in all age groups. The findings of this study suggest that CMT should be surgically corrected before the age of 3 years to obtain the best surgical mitigation effect, and the surgical release of neglected CMT is highly recommended.
